# New evidences on the regulation of SF-1 expression by POD1/*TCF21* in adrenocortical tumor cells

**DOI:** 10.6061/clinics/2017(06)10

**Published:** 2017-06

**Authors:** Monica Malheiros França, Antonio M. Lerario, Maria Candida B.V. Fragoso, Claudimara Ferini Pacicco Lotfi

**Affiliations:** I Departamento de Anatomia, Instituto de Ciencias Biomedicas, Universidade de Sao Paulo, Sao Paulo, SP, BR; II Laboratorio de Hormonio e Genetica Molecular (LIM-42), Unidade Adrenal, Divisao de Endocrinologia, Faculdade de Medicine, Universidade de Sao Paulo, Sao Paulo, SP, BR

**Keywords:** TCF21, POD1, SF-1, siRNA-*POD1*, Adrenocortical Tumor Cells

## Abstract

**OBJECTIVES:**

Transcription Factor 21 represses steroidogenic factor 1, a nuclear receptor required for gonadal development, sex determination and the regulation of adrenogonadal steroidogenesis. The aim of this study was to investigate whether silencing or overexpression of the gene Transcription Factor 21 could modulate the gene and protein expression of steroidogenic factor 1 in adrenocortical tumors.

**METHODS:**

We analyzed the gene expression of steroidogenic factor 1 using qPCR after silencing endogenous Transcription Factor 21 in pediatric adrenal adenoma-T7 cells through small interfering RNA. In addition, using overexpression of Transcription Factor 21 in human adrenocortical carcinoma cells, we analyzed the protein expression of steroidogenic factor 1 using Western blotting.

**RESULTS:**

Transcription Factor 21 knockdown increased the mRNA expression of steroidogenic factor 1 by 5.97-fold in pediatric adrenal adenoma-T7 cells. Additionally, Transcription Factor 21 overexpression inhibited the protein expression of steroidogenic factor 1 by 0.41-fold and 0.64-fold in two different adult adrenocortical carcinoma cell cultures, H295R and T36, respectively.

**CONCLUSIONS:**

Transcription Factor 21 is downregulated in adrenocortical carcinoma cells. Taken together, these findings support the hypothesis that Transcription Factor 21 is a regulator of steroidogenic factor 1 and is a tumor suppressor gene in pediatric and adult adrenocortical tumors.

## INTRODUCTION

Transcription Factor 21 (*Tcf21*, POD1, capsulin, epicardin) is a basic helix-loop-helix (bHLH) transcriptional regulatory protein that is expressed in mesenchymal cells 1 at sites of mesenchymal-epithelial interaction in the developing urogenital 2, cardiovascular, respiratory, and gastrointestinal systems 3. In the adrenal gland, POD1 is expressed exclusively in the capsule region of the adrenal cortex, as shown in mice expressing a lacZ gene reporter under the control of the regulatory region of *Pod1* 4. In the testicles of fetal mice, POD1 represses steroidogenic factor 1 (SF-1/*Nr5a1*), an orphan member of the nuclear receptor family of transcription factors required for gonadal development, sex determination and the regulation of adrenal and gonadal steroidogenesis in adult mice 5. Alterations of SF-1 dosage regulate compensatory adrenal growth after unilateral adrenalectomy, proliferation and tumorigenesis in mice 6. In humans, *SF-1* is associated with adrenocortical tumorigenesis both in children 7 and adults 8. POD1 represses *Sf-1*/*SF-1/*SF-1 expression in mouse 5, rat 9, and human adrenocortical cells 10. Moreover, *POD1* is downregulated in adrenocortical carcinoma (ACC) 10,11, melanoma 12, lung, and head and neck squamous cell carcinomas 13. In human ACC cells, we showed that POD1 binds to the *SF-1* E-box promoter sequence and inhibits *SF-1* expression and steroidogenic acute regulatory (*StAR*) expression, which is controlled by SF-1 10. However, it is unknown whether silencing the *POD1* gene promotes increased expression of the *SF-1* gene. Accordingly, here, we analyzed the expression of the *SF-1* gene after downregulation of endogenous *POD1* expression in pediatric adrenocortical tumor cells. Moreover, we verified whether POD1 overexpression causes inhibition of SF-1 protein expression.

## MATERIALS AND METHODS

### Cell cultures and cell culture transfection

The NCI-H295R adult human ACC cell line 14 and ACC-T36 10, a 6^th^–10^th^ passage secondary adult human ACC cell line, were cultured and transfected as described by França et al. 10. Cells from the ACA-T7 pediatric secondary cell line were obtained from a functioning adrenocortical adenoma (ACA) as described by Almeida et al. 15. Pediatric adenoma (weight: 10 g; stage I) was diagnosed in a 1.1-yr-old girl with mixed Cushing’s syndrome and virilization 15. The adrenocortical tumor cells were maintained at 37°C in a fully humidified 95% air-5% CO_2_ environment and cultured in Dulbecco’s Modified Eagle medium (DMEM) supplemented with 10% fetal bovine serum (FBS) and 1% penicillin/streptomycin. The experiments were performed at the 10^th^–15^th^ passage of the pediatric ACA culture cells. Briefly, 1.5 x 10^5^ ACA-T7 cells were plated into six-well tissue culture plates (Becton Dickinson Labware, Franklin Lakes, NJ, USA). After 24 h, the cells were transfected with small interfering RNA (siRNA-*POD1)* or positive or negative high CG RNAi Stealth (Invitrogen, Carlsbad, CA, USA) to a final concentration of 100 nM, combined with 9 µl of RNAiMax Lipofectamine® according to the manufacturer’s instructions (Invitrogen, Carlsbad, CA, USA).

### Total RNA extraction and qPCR siRNA (qRT-PCR)

Total RNA was extracted using TRIzol® reagent (Invitrogen) 48 h after transfection. The synthesis of cDNA and RT-qPCR analysis were performed as described by França et al. 10. A cycle threshold (Ct) value in the log-linear phase of amplification was selected for each sample in triplicate and was normalized to the β-actin expression level. Reactions were conducted in triplicate. Data were analyzed using the 2^-ΔΔCt^ method 16.

### Immunoblotting

For the protein assay, H295R and ACC-T36 cells were plated and transfected as described by França et al. 17. The cells were lysed 72 h post-transfection in RIPA buffer containing protease and phosphatase inhibitors (Sigma Aldrich Gmbh, Steinheim, Germany). The total protein concentration was determined using the Bradford assay. Total protein lysates (30 µg) were resolved by 12% SDS-PAGE and gels were blotted onto nitrocellulose membranes after electrophoresis. Non-specific binding sites were blocked for 2 h with 0.1% bovine serum albumin (BSA) or 5% non-fat dried milk in TBST (TRIS-buffered saline solution containing 1% Tween 20). All washes and antibody incubations were performed using TBST. The following primary antibodies were used: anti-SF-1 (RD Systems Inc., Minneapolis, MN, USA; 1:1000) in blocking buffer (5% non-fat dried milk in TBST) and anti-actin (1:1000) in TRIS-buffered saline containing 1% Tween 20. Proteins were visualized using enhanced chemiluminescence (ECL) detection with secondary HRP-conjugated anti-rabbit (Amersham Hybond ECL, Freiburg, Germany) or anti-mouse (Jackson ImmunoResearch Inc., West Grove, PA, USA) antibodies. Immunoblot results were quantified on a densitometer using GeneSnap and GeneTools software (SynGene-Synoptic Ltd., Cambridge, United Kingdom). Protein transfer and loading were monitored using Ponceau S staining of the membranes. The experiments were repeated in full at least three times, and SF-1 protein expression was normalized to the levels of β-actin.

### Statistical analysis

Data are presented as the mean ± standard deviation (SD) of three independent replicate experiments. Data were analyzed using the Kruskal-Wallis test (non-parametric one-way ANOVA) or paired t-tests, when indicated. The results were considered significant when *p*<0.05.

## RESULTS AND DISCUSSION

**Figure 1:**
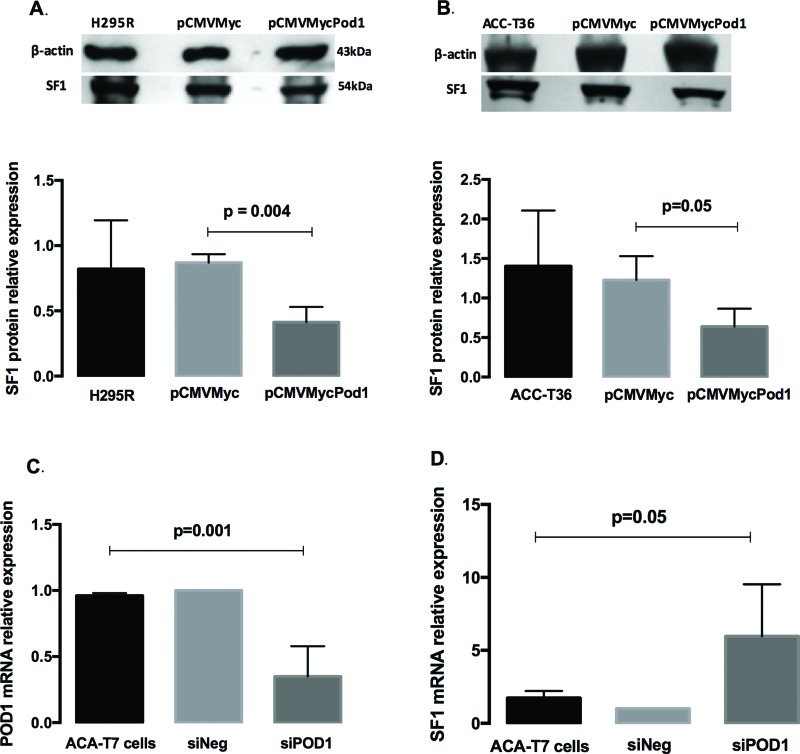
**A** and **B**: Immunoblotting analysis of the SF-1 protein levels relative to α-Actin in H295R (A) and ACC-T36 cells (B) that were transiently transfected with pCMVMycPod1 or empty pCMVMyc vectors. **C** and **D**: Quantitative reverse transcription PCR (qRT-PCR) analysis of the mRNA expression levels of *POD1* (C) and *SF1* (D) relative to β-Actin in ACA-T7 pediatric adrenocortical adenoma culture cells. The expression levels were compared using paired t-tests (A and B) or one-way ANOVA Kruskal-Wallis tests (C and D). The values represent the mean ± standard deviation of three experiments.

SF-1 protein levels in the H295R cell line (Figure 1A) decreased 0.41±0.11-fold (*p*=0.004) compared to those in the empty plasmid controls, whereas in ACC-T36 cells (Figure 1B), SF-1 protein expression decreased 0.64±0.22-fold (*p*=0.05). These results are in agreement with a previous study showing that POD1 inhibited *SF-1* mRNA expression in ACC cells 10. Next, we investigated whether knockdown of *POD1* could increase *SF-1* expression. To knockdown *POD1*, we used siRNA in ACA-T7 cells that expressed *POD1* constitutively (data not shown) and determined the mRNA levels of *POD1* and *SF-1* in ACA-T7-siRNA-transfected and ACA-T7-si*POD1*-transfected cells. *POD1* mRNA expression was significantly lower in T7-si*POD1* transfected cells (0.35±0.22-fold), with a 65% decrease compared to that in transfected controls (ANOVA, *p*=0.001; Figure 1C), whereas the mRNA levels of *SF1* increased 5.97±0.22-fold in T7-si*POD-1* cells compared to those in transfected controls (ANOVA, *p*=0.05; Figure 1D). These results support a role for POD1/*TCF21* as a regulator of SF-1 expression in adrenocortical tumor cells. Considering that POD1 is a repressor of SF-1 expression, that increased SF-1 dosage can trigger human adrenocortical cell proliferation 18, and that SF1 amplification 19 and SF1 overexpression 7 are characteristic of childhood adrenocortical tumors, the results of our study improve the knowledge of how the tumorigenic process is controlled in adrenocortical tumor cells. Taken together, the findings of this study and the POD1 inactivation observed in several types of tumors suggest that POD1 may act as a tumor suppressor in pediatric and adult adrenocortical tumors.

## AUTHOR CONTRIBUTIONS

Lotfi CF conceived the project. Lotfi CF and França MM designed the experiments. Lotfi CF, França MM, Lerario AM and Fragoso MC analyzed the data. França MM performed the experiments. França MM and Lotfi CF wrote the manuscript.
